# End Stage Kidney Disease in Non-citizen Patients: Epidemiology, Treatment, and an Update to Policy in Illinois

**DOI:** 10.1007/s10903-021-01303-7

**Published:** 2021-11-13

**Authors:** Brittney S. Lange-Maia, Tricia J. Johnson, Yumiko I. Gely, David A. Ansell, J. Kevin Cmunt, Elizabeth B. Lynch

**Affiliations:** 1 Department of Preventive Medicine, Rush University Medical Center, Chicago, IL USA; 2 Center for Community Health Equity, Rush University Medical Center, Chicago, IL USA; 3 Department of Health Systems Management, Rush University, Chicago, IL USA; 4 Rush Medical College, Rush University Medical Center, Chicago, IL USA; 5 Department of Internal Medicine, Rush University Medical Center, Chicago, IL USA; 6 Gift of Hope Organ and Tissue Donor Network and Illinois Transplant Fund, Itasca, IL USA; 7 Rush University Medical Center, 1750 W. Harrison St. Suite 1000, Chicago, IL 60612 USA

**Keywords:** Kidney transplant, End-stage kidney disease, Health disparities, Immigrant health

## Abstract

End-stage kidney disease (ESKD) is common in the U.S. There is no cure, and survival requires either dialysis or kidney transplant. Medicare provides coverage for most ESKD patients in the U.S., though non-citizens are excluded from most current policies providing standard ESKD care, especially regarding kidney transplants. Despite being eligible to be organ donors, non-citizens often have few avenues to be organ recipients—a major equity problem. Overall, transplants are cost-saving compared to dialysis, and non-citizens have comparable outcomes to the general population. We reviewed the literature regarding the vastly different policies across the U.S., with a focus on current Illinois policy, including updates regarding Illinois legislation which passed in 2014 providing non-citizens to receive coverage for transplants. Unfortunately, despite legislation providing avenues for transplants, funds were not allocated, and the bill has not had the impact that was expected when initially passed. We outline opportunities for improving current policies.

## Introduction

End-stage kidney disease (ESKD) is common in the U.S. There is no cure, and either dialysis or kidney transplant are crucial for survival. Policy changes in the 1970s allowed Medicare coverage for patients with ESKD and were instrumental in improving outcomes for patients with ESKD in the U.S. overall. However, non-citizens—particularly undocumented patients—are excluded from most policies and programs providing coverage for ESKD care. Policies related to caring for non-citizens with ESKD vary dramatically between states, further contributing to unequal access. Unique legislation passed in Illinois in 2014 provided avenues for non-citizens to obtain transplants [1]. However, state budget issues and leadership changes hindered the effectiveness of this legislation, and it did not improve equity as intended. The purpose of this paper is to (1) provide an overview of the epidemiology of ESKD and inequities; (2) describe treatment options for ESKD and policies impacting options and (3) discuss policies and treatment options for non-citizen patients and their impacts, with an update on Illinois policy. Potential new legislation could provide permanent solutions to access barriers and serve as a model for other states.

## Part 1: End Stage Kidney Disease (ESKD) Treatment and Policy

### ESKD Epidemiology

Approximately 14.8% of U.S. adults ages 20 years and older have chronic kidney disease (CKD), equating to approximately 30 million Americans [2, 3]. However, nearly 9 out of every 10 adults with CKD are unaware that they have it, as the disease is primarily asymptomatic until late stages [2]. Progression can be slowed with early treatment, and early treatment can also help to manage complications.

ESKD describes patients with irreparable organ damage who require kidney replacement therapy, and this commonly occurs when kidney function is below 15%. Approximately 785,883 patients were treated for ESKD in the U.S. in 2018, and Illinois has the third highest prevalence of ESKD in the country, behind Washington D.C. and South Dakota [2, 4, 5]. While ESKD is associated with high morbidity and mortality, kidney transplants significantly improve survival. Five-year survival for patients on dialysis is only 35%, compared to 80% for transplant recipients [5]. Stark racial and ethnic differences exist in the incidence of ESKD; non-Hispanic Black adults have 2.7 times higher incidence rates of ESKD compared to non-Hispanic Whites, and Hispanics have about 1.3 times higher incidence of ESKD compared to non-Hispanic Whites [5].

### ESKD Treatment

Two primary treatment options exist for ESKD: (1) dialysis and (2) kidney transplants. More than 90% of dialysis patients are on hemodialysis, which involves about 3–5 hour treatments three times per week, placing a significant burden on patients and their families. Unless they receive a kidney transplant, patients undergoing dialysis generally need to continue regular treatments indefinitely. A year of hemodialysis costs the Medicare system approximately $91,800 per patient, and life expectancy on dialysis is 5–10 years [6].

Kidney transplantation is the optimal treatment for ESKD. In contrast to dialysis, Medicare spends only $35,800 annually for each transplant patient after the initial cost of the transplant—which can be upwards of $100,000 [5]. Ultimately, kidney transplants are cost-effective relative to lifetime dialysis due to both lower medical costs and improved outcomes over the long term. Patients are often able to return to work after kidney transplant and quality of life can significantly improve [7–10]. Compared to hemodialysis, patients who have undergone a kidney transplant have better physical function, are more engaged in social and recreational activities, are better able to continue working, and report greater independence [11].

Approximately 30% of ESKD patients in the U.S. are living with a functioning transplanted kidney [4]. Most patients receive organs from a deceased donor, though many patients rely on receiving a kidney from a living donor. Living donor transplants last about 15–20 years, whereas transplants from a deceased donor last 10–15 years [12]. Patients waiting for a deceased donor typically wait 3–5 years [12].

### Policy Regarding ESKD Treatment Coverage

Disparities in ESKD—particularly by socioeconomic status—have been pervasive for decades. Until the early 1970s, ESKD survival was highly dependent on socioeconomic factors due to high treatment costs [13]. The enactment of legislation in 1972 allowed qualifying patients with ESKD to enroll into Medicare, even if they were otherwise ineligible due to age [14]. This was the first Medicare provision that expanded coverage based on a specific medical condition [13]. ESKD coverage through Medicare has provided lifesaving care for over a million patients and is considered one of the most successful federal health programs in the U.S. [13]. In 2015, 81% of patients with ESKD received health insurance coverage through Medicare [15]. However, even with Medicare coverage, the out-of-pocket costs from co-payments and deductibles are often substantial for patients without an additional source of health insurance coverage.

Long-term success of a kidney transplant necessitates ongoing treatment with immunosuppressive drugs, which require a 20% coinsurance through Medicare Part B [13]. Until recently, Medicare coverage for ESKD patients (unless they qualified for Medicare due to age or disability) would end either 3 years after kidney transplant or 12 months after dialysis stopped. However, in 2020 the Comprehensive Immunosuppressive Drug Coverage for Kidney Transplant Patients Act was passed, removing the 3-year immunosuppressive drug coverage limitation [16]. It is estimated that the updates to U.S. policy removing the time limit for immunosuppressive drug coverage will prevent approximately 375 graft failures on an annual basis [16, 17].

## Part 2: Gaps in Current ESKD Policies and Impacts for Non-citizens

Despite providing coverage for the vast majority of ESKD patients, Medicare is not universal and excludes non-citizens. Further, some patient groups, particularly non-citizens without qualifying status, do not qualify for Marketplace coverage through the Affordable Care Act either, further limiting their options. Given their lack of health insurance coverage, many non-citizen patients—and particularly patients who are undocumented—don’t receive care until they develop severe symptoms of the disease and need dialysis, and therefore, seek medical care through an emergency department. The 1986 Emergency Medical Treatment and Active Labor Act (EMTALA) requires that emergency medical care be provided to anyone, regardless of their ability to pay [18]. Any patient coming to an emergency department with an emergent medical condition must be provided with treatment, such as dialysis, until stable or transferred to another hospital. Without access to Medicare coverage or access to private health insurance coverage through the Marketplace, non-citizen patients must rely on emergency departments, charity care or state-funded sources, such as Medicaid, for treatment.

### Dialysis Options for Hemodialysis Vary by State

Coverage of dialysis treatment for non-citizens, and particularly undocumented patients, depends on state policy and local safety nets, thus varying widely across states [19]. States have discretion in defining what constitutes a medical emergency and which treatments are reimbursed. Some states have emergency Medicaid policies that cover outpatient hemodialysis services, but in many states, patients must present to an emergency department in critical condition to receive hemodialysis–referred to as emergency-only, or emergent hemodialysis. In these states, emergency-only dialysis is the only option for many undocumented patients [19]. Emergency-only dialysis is an expensive way to deliver dialysis and has worse outcomes than standard-of-care scheduled dialysis [20]. Standard-of-care hemodialysis treatment includes placement of a permanent vascular access, using either an arteriovenous fistula or an arteriovenous graft, and regular sessions in a dialysis center. Patients receiving emergency-only dialysis rarely receive this standard of care due to lack of medical coverage [3, 21]. Emergency dialysis is usually initiated without an arteriovenous fistula or an arteriovenous graft, resulting in a greater risk of infection and mortality [22]. Patients receiving emergency-only dialysis versus outpatient dialysis have 5 times higher 3-year mortality rates, and 14 times higher 5-year mortality rates [3]. Further, acute care hospital days are almost 10 times higher for patients who receive emergency-only dialysis compared to patients receiving standard dialysis [3].

As of March 2019, only 12 states (including Illinois) allowed for Medicaid reimbursement of standard outpatient dialysis for undocumented patients; 16 states explicitly prohibited Medicaid reimbursement for standard outpatient dialysis for undocumented patients, although other funds like state risk pools and health system charity care may exist; and the remaining 22 states had no provision for undocumented patients, thus subjecting them to emergency-only dialysis [19]. To make matters worse, in many states non-citizen and undocumented patients are not eligible for emergency-only dialysis until there are life threatening signs and symptoms of kidney failure that could result in death unless imminently treated [23]. Undocumented patients who have standard dialysis have the same outcomes as the overall U.S. CKD/ESKD population, but mortality is twice as high for undocumented patients receiving emergency-only dialysis [3].

### Patient Experiences with Emergency-Only Hemodialysis

In addition to being inefficient in terms of cost and poorer quality of care, the experience of emergency-only hemodialysis is traumatizing for patients, caregivers, their families, and healthcare personnel providing care. Patients relying on emergency-only dialysis must meet threshold lab values to be eligible to receive treatment at the emergency department, and these thresholds may vary by institution and by how many other patients are waiting for dialysis [23, 24]. Patients not meeting thresholds are turned away without receiving treatment. To avoid this, patients report waiting until symptoms are severe before presenting to the emergency department, putting themselves at risk of death on a regular basis [24]. Patients reported frequent near-death and resuscitation experiences, resulting in extreme anxiety and insomnia for themselves and their families [24]. Further, emergency-only hemodialysis patients do not receive standard of care medication for nausea, vomiting, and dyspnea [24].

Stress on families is severe, and caregivers report high levels of psychosocial distress and caregiver burnout due to emergency-only dialysis policies, particularly when a patient is denied dialysis [24, 25]. This impacts the caregiver’s health and well-being and ability to work or complete schooling due to the heightened unpredictability of ESKD when emergency dialysis is the only option available [25].

Providing emergency-only hemodialysis also contributes to moral distress and professional burnout among clinicians. In a study examining perspectives of clinicians who provide care to undocumented immigrants in safety-net systems, emotional exhaustion, anguish, and feelings of jeopardizing patient trust were commonly reported [23]. They saw the practice of emergency-only hemodialysis as inefficient, a poor use of resources, and as propagating injustice as it “reflects an overtly different and inferior standard of medical care based upon nonmedical criteria” [23].

Patients transitioning from emergency-only hemodialysis to scheduled dialysis experience improvements in quality of life, as well as improvements in symptoms [26]. Patients report relief due to the ability to receive consistent care, a renewed sense of hope, and immediate health gains. Still, transitioning from emergency-only to scheduled dialysis comes with its own challenges for patients, including anxiety about navigating systems related to dialysis care, concerns about cost and insurance, increased need for reliable transportation to attend dialysis sessions, and potential work disruptions due to having more dialysis sessions. Patients also fear discrimination due to limited English proficiency and their legal status in the U.S. [26]. Policy changes that allow patients to move from emergency-only to scheduled hemodialysis should be accompanied with clear communication to patients regarding processes and costs and safeguards regarding immigration status.

### Despite Promise of Reduced Costs, Transplant Options are Limited for Undocumented Patients

There is no written nationwide or state policy regarding transplants for undocumented individuals; instead, transplant centers individually decide eligibility. Lack of insurance for transplant costs and aftercare effectively excludes undocumented patients, even though transplants are generally cost saving relative to dialysis. These policies—or lack thereof—create a double standard regarding organ transplantation. Undocumented patients (and others unable to obtain health insurance) are eligible to be organ donors, but few routes exist for them to be organ recipients.

Undocumented patients are excluded from transplantation even though they are often good transplant candidates. Relative to other kidney patients, undocumented patients tend to be healthier, younger, and more likely to have access to living donors [27]. A recent study compared characteristics of undocumented patients and U.S. residents receiving outpatient dialysis treatments covered by New York State emergency Medicaid funds and found that undocumented patients were younger and healthier relative to the legal residents, with lower rates of comorbidities[28]. Undocumented patients were also more likely to be working, as only 6% of the U.S. resident patients were employed, but in contrast, 51% of undocumented patients were still working and most (83%) were working full-time. Of those who were working, 100% said they would continue working if they received a transplant. Furthermore, 52% of undocumented patients who had stopped working had left their job due to their kidney disease and the intensive dialysis schedule [28]. Overall among working-age patients with ESKD, only about 36% are able to work [29].

### Ethical Issues Regarding Transplants for Non-citizens

There are ethical considerations in allocating organs for transplant, particularly since the demand for kidneys exceeds availability. While the lack of pathways for becoming an organ recipient—in spite of qualifying to be organ donors—and unequal treatment due to policies rather than medical need are two major ethical issues for noncitizens, policymakers are also faced with other ethical considerations. We outline some of the most common ethical issues.

Critics of providing organ transplants to undocumented patients argue that undocumented patients face additional barriers to post-transplant care that threaten graft survival, including the possibility of deportation from the United States and lack of access to follow-up care. Research findings do not support this argument, and in fact, have shown that undocumented patients have lower risk of transplant loss compared to U.S. citizens [30]. In a study comparing transplant outcomes between U.S. citizens, permanent residents, and patients who were undocumented, the undocumented patients were younger, had better functional status, and had fewer comorbid conditions [30]. After adjusting for demographic characteristics, dialysis factors, transplant factors, and comorbid conditions, undocumented patients had approximately 33% lower risk of graft loss compared to patients who were U.S. citizens. Further, opponents of non-citizens, and especially undocumented patients, receiving organ donations argue that these individuals do not contribute to public systems in the U.S., and therefore, should not be eligible for an organ transplant. In actuality, undocumented immigrants contribute approximately $11.74 billion in local, state, and federal taxes [31]. Despite these contributions, undocumented immigrants are unable to qualify for benefits, aside from some state programs.

Another argument cited by critics is that the inclusion of non-citizens on the transplant waiting list exacerbates the shortage of donor organs. However, research using national data from the United States Renal Data System that include nearly all kidney transplants in the United States has shown that undocumented patients are more likely to have a living donor compared to U.S. citizens and permanent residents (40% versus 32% versus 27% respectively) [30]. Another study of undocumented patients undergoing dialysis found that 60% had at least one potential living donor [28]. The undocumented population does participate in deceased organ donation as well. Denial of access to transplantation for a population that is asked to donate is inherently unethical. The fact that this population likely provides more organs for transplant than they receive (due to demographics and consent for donation patterns) counters the argument that allowing this group to participate in transplantation will reduce the overall number of organs available to other populations.

## Part 3: Illinois and Transplants for Non-citizens

### Illinois Legislation: Senate Bill 741

In 2014, Illinois passed Illinois Senate Bill (SB) 741, becoming one of the first states providing medical coverage for kidney transplants for non-citizens. SB741 allowed Illinois to extend Medicaid coverage to newly eligible low-income adults, federal Medicaid funding under the Affordable Care Act. Importantly, this legislation also allowed for kidney transplant coverage (section 5-5e) to non-citizens receiving dialysis through the state-funded program, noting that transplants would be cost effective when compared to dialysis, and would ultimately decrease costs for Illinois’ emergency dialysis program [1, 32]. This provision was met with strong bipartisan support. Around that same time, Illinois passed SB957, allowing undocumented immigrants to obtain a state-issued driver’s license. Recipients of these new drivers’ licenses were encouraged to become organ donors.

The provision for kidney transplants to non-citizen patients includes the following restrictions: (1) the transplant benefit is available only to uninsured patients with ESKD who are in the Medicaid emergency dialysis program, and (2) it excludes CKD patients who have not yet started dialysis. The transplant benefit is administered by the Illinois Department of Healthcare and Family Services and is paid from state public aid funds to transplant centers and covers all pre-transplant, donor, and post-transplant costs, although a separate pharmacy benefit covers the costs of immunosuppressive medications. Unfortunately, the level of reimbursement for kidney transplants through this program was low and insufficient to cover transplant costs and after care. At the time, transplant advocates were working to clarify the legislation, but unfortunately these issues were not resolved, and budgetary complications arose making this legislation ineffective.

### Fallbacks of this Legislation and Role of Non-profit Organizations

Though this legislation was a breakthrough in terms of creating potential avenues for transplants for undocumented patients, it ultimately did not result in actual practice changes or improve equity on its own. First, programs arising from Senate Bill 741 relied on ongoing budgetary support, which ceased in 2015 with the Illinois state budget crisis. Further, instead of utilizing emergency dialysis through the State, a provision of the program, many non-citizen patients instead obtain coverage through the American Kidney Fund (AKF). AKF covers health insurance premiums that cover scheduled dialysis. AKF allows access to dialysis but coverage assistance ceases at the end of the year once a patient receives a transplant. Thus, AKF covers care leading up to and including a transplant, but not extended care and medications after the transplant, which is crucial for transplant survival.

Given the gaps in existing legislation and programs, the Illinois Transplant Fund (ITF) was formed to provide crucial transplant access and follow-up transplant care. The ITF covers health insurance premiums for people who are unable to pay and do not qualify for other financial insurance subsidies or government insurance programs, ensuring patients can demonstrate proof of long-term access to post-transplant medications. ITF covers 100% of monthly health insurance premiums for at least 36 months post-transplant, gradually transitioning to patient responsibility in the fourth and fifth years post-transplant. Patients who are unable to transition for financial reasons and continue to qualify have continued to be covered by the ITF. ITF fills an important gap in care during this post-transplant period, which is vital to prevent transplant failure.

ITF was established in 2015 by the Gift of Hope Organ and Tissue Donor Network, Illinois’ organ procurement agency. ITF provides a non-government funded approach to grant access to transplants for the uninsured. The aim is “to increase access to organ transplants by targeting the inequity of health insurance access, helping to eliminate this barrier to life-saving organ transplant surgery” [33]. Funding for the ITF comes from private donations, including Chicago-area academic medical centers. Since its inception, the ITF has successfully supported over 200 patients in their transplant process, despite enormous increases in insurance premium costs over the last 5 years. Patients who are supported by the ITF have a higher rate of living donors (40%) than the national average (37%) from the 2015–2020 time frame. Even after accounting for an initial higher cost in the year of the transplantation, it is estimated that ITF support saves the health care system roughly $50,000 per patient per year, or currently over $10 million per year.

### Future Directions

Illinois has made tremendous progress in eliminating inequities in transplant access through a combination of State support and private nonprofit programs. However, the sustainability of the programs are always an issue and a more permanent solution would best serve the community. Possible changes are as follows:

1. Revise regulations implementing SB741 (state reimbursement of transplant costs for patients on emergency dialysis) to reimburse organ transplant costs at the level of reimbursement by Illinois Medicaid.
2. SB2294 was signed into law on July 6, 2021 creating a benefit for non-citizen kidney transplant recipients to obtain coverage for immunosuppressive drugs and posttransplant care. Regulators need to implement the law and ensure that reimbursement is reasonable and coverage is comprehensive. While the state has allowed for coverage of kidney transplant recipients, an improvement would be to expand SB2249 to nonrenal organ transplant recipients.
3. Expand the Illinois State Chronic Renal Disease Program to include non-citizens. Currently, eligibility requirements for this program state that patients need to be U.S. citizens or meet immigration requirements to receive support; however, if this program could be expanded to support non-U.S. citizens, collaborations could help further bridge the gap of this health inequity and allow more undocumented and uninsured patients to receive standard of care treatment [34]. To properly support these changes, potential options for funding should be explored. One potential option is public private partnerships (PPPs). PPPs require specific legislative support to coordinate private and public resources to provide certain resources to the public, including working with current nonprofits. PPP funding could be further explored to allocate a percentage of support from both private and public funds to support more patients in gaining access to a transplant.

## Conclusion

In short, ESKD is a common illness requiring extensive medical care, with either dialysis or kidney transplant being crucial for survival. Though most ESKD patients in the U.S. have access to care through Medicare, some patients remain left out, particularly non-citizens and especially undocumented patients. We have outlined policy differences between states and have highlighted policies in Illinois. Though Illinois allows for treatment options left out of other states, further work is needed to strengthen these policies, which would improve patient care, reduce costs, and close crucial equity gaps.
